# Mouse models of diffuse large B cell lymphoma

**DOI:** 10.3389/fimmu.2023.1313371

**Published:** 2023-12-06

**Authors:** Areya Tabatabai, Aastha Arora, Svenja Höfmann, Maximilian Jauch, Bastian von Tresckow, Julia Hansen, Ruth Flümann, Ron D. Jachimowicz, Sebastian Klein, Hans Christian Reinhardt, Gero Knittel

**Affiliations:** ^1^ Department of Hematology and Stem Cell Transplantation, University Hospital Essen, West German Cancer Center, German Cancer Consortium Partner Site Essen, Center for Molecular Biotechnology, University of Duisburg-Essen, Essen, Germany; ^2^ Department I of Internal Medicine, University of Cologne, Faculty of Medicine and University Hospital Cologne, Center for Integrated Oncology Aachen Bonn, Cologne, Germany; ^3^ Center for Molecular Medicine, University of Cologne, Cologne, Germany; ^4^ Cologne Excellence Cluster on Cellular Stress Response in Aging-Associated Diseases (CECAD), University of Cologne, Cologne, Germany; ^5^ Mildred Scheel School of Oncology Aachen Bonn Cologne Düsseldorf (MSSO ABCD), Faculty of Medicine and University Hospital of Cologne, Cologne, Germany; ^6^ Max Planck Institute for Biology of Ageing, Cologne, Germany

**Keywords:** diffuse large B cell lymphoma (DLBCL), genetically engineered (GE) animals, lymphoma, animal models, mouse models

## Abstract

Diffuse large B cell lymphoma (DLBCL) is a genetically highly heterogeneous disease. Yet, to date, the vast majority of patients receive standardized frontline chemo-immune-therapy consisting of an anthracycline backbone. Using these regimens, approximately 65% of patients can be cured, whereas the remaining 35% of patients will face relapsed or refractory disease, which, even in the era of CAR-T cells, is difficult to treat. To systematically tackle this high medical need, it is important to design, generate and deploy suitable *in vivo* model systems that capture disease biology, heterogeneity and drug response. Recently published, large comprehensive genomic characterization studies, which defined molecular sub-groups of DLBCL, provide an ideal framework for the generation of autochthonous mouse models, as well as an ideal benchmark for cell line-derived or patient-derived mouse models of DLBCL. Here we discuss the current state of the art in the field of mouse modelling of human DLBCL, with a particular focus on disease biology and genetically defined molecular vulnerabilities, as well as potential targeting strategies.

## Introduction

Malignant lymphomas constitute a highly diverse spectrum of diseases that originate from various distinct stages of lymphocyte development. Collectively, they rank as the sixth most common cancer entity (1–3). Lymphomas are further divided into Hodgkin- (HL) and non-Hodgkin lymphomas (NHL) (1). Among the NHLs, cases derived from B- and T cells are distinguished (1). Additionally, lymphomas can further be subcategorized based on their clinical features, classifying them as either indolent or aggressive lymphomas (1). This review exclusively focusses on aggressive B-NHL and will only discuss indolent lymphomas when these entities provide critical information relevant for our understanding of the overall disease biology. We will also omit an in-depth discussion of Burkitt lymphoma models, to allow enough space for a more detailed review of diffuse large B cell lymphoma (DLBCL) models.

The majority of aggressive lymphomas originate from the germinal center (GC) - a highly specialized immunological structure - which transiently forms upon antigen encounter of a naïve B cell in the context of a T cell-dependent adaptive immune response. This specialized anatomic structure serves to facilitate B cell receptor (BCR) affinity maturation and class switch recombination (4). In line with their GC origin, Burkitt lymphomas (BL), follicular lymphomas (FL) and diffuse large B cell lymphomas (DLBCL) are GC-experienced, as evidenced by somatic hypermutation (SHM)-mediated mutagenesis within their BCR variable regions – an irreversible marker of GC passage (5–8). The functional output of the GC reaction is the development of a population of plasma cells capable of secreting high-affinity antibodies neutralizing the initially recognized antigen, as well as the production of memory B cells, which possess the ability to rapidly differentiate into antibody-secreting plasma cells upon antigen re-exposure.

While the generation of memory B cells and plasma cells is of critical importance for efficient control of infections and thus for survival, the GC reaction, due to its unique biological features, also poses the risk of lymphoma development. GCs are established upon antigen encounter and T cell co-stimulation through which B cells are activated via B cell receptor (BCR)-, CD40- and Toll-like receptor (TLR) signaling. Altogether, this signaling input triggers NFκB activation and the subsequent expression of genes mediating B cell activation and proliferation to drive GC initiation (9, 10). The central regulator of the GC response is the transcriptional repressor BCL6. Once GCs are established upon antigen encounter, BCL6 orchestrates the GC reaction through the repression of numerous genes involved in distinct biological processes, including apoptosis, DNA damage response and genome maintenance, cell cycle checkpoint signaling, as well as plasma cell differentiation (4, 11, 12).

Particularly the transient repression of DNA damage response programs in conjunction with the ability to override cell cycle checkpoints and the attenuation of the apoptotic cell death machinery, enable dark zone B cells to establish a hyper-proliferative program. This growth acceleration is associated with an extraordinarily high risk of accumulating oncogenic aberrations. These genomic lesions can manifest as single nucleotide variants resulting from off-target Activation Induced Cytidine Deaminase (AID)-mediated deamination events, as well as structural variations through erroneous AID-mediated class-switch recombination (3, 5, 13–17). GC B cells can undergo repeated transitions between the hyperproliferative dark zone compartment and the light zone, where they compete for antigen presented on follicular dendritic cells and T cell help provided by T-follicular helper cells (5, 12, 14). BCL6 further facilitates the retention of GC B cells within the GC reaction by preventing the induction of plasma cell differentiation programs (5, 12, 14). Only upon signal integration from the BCR, CD40, BAFF and TLRs can high-affinity GC B cells accumulate a robust NFkB signal, which drives IRF4 expression leading to BCL6 silencing and subsequent BLIMP1 expression, ultimately terminating the GC program and facilitating post-GC differentiation (10, 18, 19).

In summary, the GC reaction serves a critical purpose in diversifying the BCR repertoire and generating high-affinity antibodies to eradicate infectious agents. However, this immense potential for affinity maturation comes at the cost of a significant risk of accumulating lymphoma-promoting genomic aberrations. Hence, it is perhaps not surprising that the majority of mature B-NHLs originate from GC B cells, as evidenced by these lymphomas typically displaying genomic signatures indicative of GC experience, such as hypermutated immunoglobulin variable regions or the expression of a class-switched constant region. As diffuse large B cell lymphoma is the most common lymphoma, this review will primarily focus on this entity and the associated molecularly defined subtypes. As this topic has been reviewed previously (5, 14), we primarily focus on novel alleles that have recently emerged.

Given the extraordinary architectural and biological complexity, as well as the dynamic nature of the GC, it is perhaps not surprising that sufficient *in vitro* models of this lymphoid structure are still lacking. Activation and expansion of naïve B cells *ex vivo* can be achieved by stimulation of the Toll-like receptor pathway with LPS or CpG (20). Several combinations of CD40 and BCR stimulation by activating antibodies alone or together with IL-4 and/or IL-21 treatment result in B cell expansion and generation of cells with memory B or plasma cell phenotypes (20–22). Fibroblasts stably expressing BAFF and CD40L have been used for the *in vitro* generation of GC B cells that then differentiate into memory B or plasma cells (23). B and T cells isolated from genetically engineered mice expressing ovalbumin-specific B cell- and T cell receptors can be employed *in vitro* as a model system recapitulating phagocytic antigen uptake (23). While these systems are able to mimic B cell expansion and plasma cell/memory B cell differentiation to some extent, they fail to recapitulate the cellular complexity of the GC response with T_FH_ cells, follicular dendritic cells and B cells as the main participants. Additionally, the compartmentalization of the GC into the dark and light zone is not well-represented in cell culture models, which generates a temporal and spatial profile of stimuli provided to the B cell. Lastly, different stimulus combinations (BCR, TLR, CD40, cytokines) prompt B cells to proliferate *in vitro*, but potentially differ from stimulus intensities *in vivo*. Thus, genetically engineered mouse models (GEMMs) of GC-derived lymphomas have played, and will likely continue to play, an important role in unraveling the intricate GC biology and lymphomagenesis. However, when conceptualizing and designing proper *in vivo* modeling experiments, it is of utmost importance to have a robust understanding of human disease biology, as our ultimate goal is to model the human scenario. Thus, prior to discussing lymphoma GEMMs, it is appropriate to briefly review the genomic complexity of human DLBCL.

Traditionally, DLBCL has been subdivided according to transcriptome-based clustering into germinal center B cell-like (GCB) and activated B cell-like (ABC) DLBCL (4, 24, 25). This classification, rooted in the cell of origin (COO), distinguishes subtypes with distinct biology, pathogenesis and clinical response to frontline chemo-immune therapy (4, 26, 27). In addition to transcriptome-based subtyping, two independent comprehensive genomic analyses recently provided a framework for a molecular subtyping algorithm for DLBCL (2, 3). The Dana-Farber group defined 5 DLBCL clusters (3). These clusters were defined by 1) *BCL6* structural variants in combination with *NOTCH2* aberrations (C1), 2) Bi-allelic *TP53* inactivation (*TP53* mutations and *17p* copy number losses) in combination with haploinsufficiencies of *9p21.13/CDKN2A* and *13q14.2/RB1* (C2), 3) *BCL2* mutations with concordant *BCL2* structural variants in combination with *EZH2*-, *CREBBP*- and *KMT2D* mutations and additional activating alterations of the PI3K pathway (C3), 4) mutations in linker and core histone genes in combination with aberrations in immune evasion molecules, NFkB and RAS/JAK/STAT signaling molecules (C4) and 5) *18q* gains, likely affecting *BCL2* and/or *MALT1* in combination with *MYD88*- and *CD79B* mutations, as well as lesions that enforce a plasma cell differentiation block (aberrations in *TBL1XR1*, *PRDM1* and *SPIB1*)(C5 DLBCL). Notably, subgroups associated with a particularly high risk of treatment failure include C2, C3 and C5. In a parallel approach, the NCI group employed a supervised approach, allowing the classification of ~50% of the cases into four genetically defined DLBCL subtypes with substantial overlap with these clusters (2). This approach enabled the clustering of lymphomas with co-occurring *MYD88*- and *CD79B* mutations (MCD), *BCL6* rearrangements and *NOTCH2* mutations (BN2), *EZH2* mutations and *BCL2* rearrangements (EZB), as well as *NOTCH1* mutations (N1) (2). More recent work of the same group extended this classification system by two additional clusters, defined by aneuploidy together with *TP53* inactivating mutations (A53), as well as mutations in *SGK1* cooccurring with *TET2* lesions (ST2) (28).

While anthracycline-based first-line chemo-immune therapy regimens achieve cure rates of approximately 65% in patients with DLBCL, relapsed or refractory disease remains a major clinical challenge. Until recently, the standard of care in second-line was confined to either intensive salvage therapy followed by high-dose consolidation and autologous stem cell rescue, or a number of palliative regimens for patients deemed ineligible for intensive consolidation. However, chimeric antigen receptor (CAR) T cells are currently revolutionizing the therapeutic landscape in r/r DLBCL, and anti-CD19 CAR-T cells are now firmly established in second and third-line treatment algorithms, substantially reducing the need to deploy high-dose chemotherapy regimens (29). While these products appear to be curative in approximately 35-40% of patients, representing a significant advancement in our treatment strategies, the majority of r/r DLBCL patients treated with CAR-T cells still do not experience long-term benefit. Thus, establishing pre-clinical models that faithfully recapitulate central aspects of DLBCL subtype-specific disease biology *in vivo*, to serve as experimental platforms for the conceptual development of novel therapeutic approaches, remains a major goal in translational lymphoma research.

## *Cre* alleles with relevance to DLBCL modeling

Throughout the recent years, numerous highly relevant mouse models of aggressive lymphoma have been generated, to mimic critical features of the human disease with ever increasing precision. This development has been fueled in large parts by our expanding knowledge of recurrent genomic aberrations in human lymphomas, as well as an understanding of the molecular dialogue between lymphoma cells and cellular components of their microenvironment, as well as immune surveillance mechanisms. In parallel to this enhanced understanding of human disease biology, which serves as a guiding principle in GEMM design, technology developments in gene targeting and the availability of multiple highly specific recombinase tools have further catalyzed the development of mouse models that faithfully mimic the human disease. These genome editing tools include the Cre/loxP, Flp/FRT and Dre/Rox recombinase systems and the integrase system PhiC31/attB/attP (30). Inducibility of these recombinase systems can be achieved by fusing the recombinases to a modified estrogen receptor (ER) ligand binding domain, which leads to cytoplasmic retention of the fusion protein in the absence of the tamoxifen. Only upon tamoxifen binding, the recombinase fusion protein enters the nucleus to mediate DNA recombination. Inducibility can further be achieved through recombinase fusions to the progesterone receptor, which allows activation via the progesterone analog RU-486, or through dihydrofolate reductase (DHFR) fusions, which enable trimethoprim-induced stabilization of the recombinase fusion protein (31–33). Lastly, photoactivatable recombinases have been developed, recently (31). Altogether, these tools allow researchers to mimic inducible multistep tumorigenesis through the use of dual or triple recombinase strategies. The recent development of CRISPR/Cas9 or dCas9 technology, which is also available in a conditional fashion, for instance through a *Rosa26^Lox-STOP-Lox.Cas9-P2A-EGFP^* allele (34), has further increased our ability to precisely edit the murine genome.

In the context of B-NHL modeling, a number of *Cre* alleles have been developed. These include *Cd19^Cre^*, where the *Cre* expression cassette was targeted into exon 2 of the endogenous *Cd19* allele (35). Similarly, to create an inducible *Cre* allele, an ERT2-fused *Cre* cDNA was targeted into exon 2 of the *Cd19* locus, yielding a *Cd19^CreERT2^* allele (36). The *Mb1^Cre^* allele was generated by replacing exons 2 and 3 of the *Cd79a* gene with a codon-optimized *Cre* (37). The ATG Start codon of *Cd79a* exon 1 was deleted, leading to abolished endogenous *Cd79a* expression (37). Both, *Mb1^Cre^* and *Cd19^Cre^* are expressed at early B cell developmental stages, enabling Cre-mediated genome editing throughout B cell development. In contrast, the bacterial artificial chromosome (BAC) transgenic *Cd21^Cre^* allele is expressed when transitional B cells differentiate into mature long-lived peripheral B cells (38). In addition to these alleles, the *Cγ1^Cre^* and *Aicda^Cre^* alleles allow recombination at the (pre-)germinal center stage of B cell development. The *Cγ1^Cre^* allele was generated by introducing *Cre* cDNA preceded by an internal ribosomal entry site into the 3’ region of the *Cγ1* locus upstream of an internal polyadenylation site, essentially leading to co-expression of the *Cγ1* germline transcript and *Cre* from the endogenous *Cγ1* locus (39). While CSR has been regarded a hallmark of the germinal center process, recent data suggests that germline transcript expression peaks already prior to GC entry in a pre-GC B cell population that is primed for CSR before entering the germinal center (40, 41). Expression of *Aicda* has been detected 12h after the induction of germline transcript expression, 24 hours before the appearance of GC B cells and peaks during the GC reaction (41). The *Aicda^Cre^* allele was generated by targeting *Cre* cDNA into exon 1 of the *Aicda* locus (42). Next to a constitutive *Aicda^Cre^* allele, an *Aicda^CreERT2^* allele was generated by targeting exon 2 of the *Aicda* locus leading the expression of an ERT2-fused Cre recombinase, which also contains the first four N-terminal amino acids of the AID protein (43).

## Autochthonous mouse models of diffuse large B cell lymphoma

**Figure 1:**
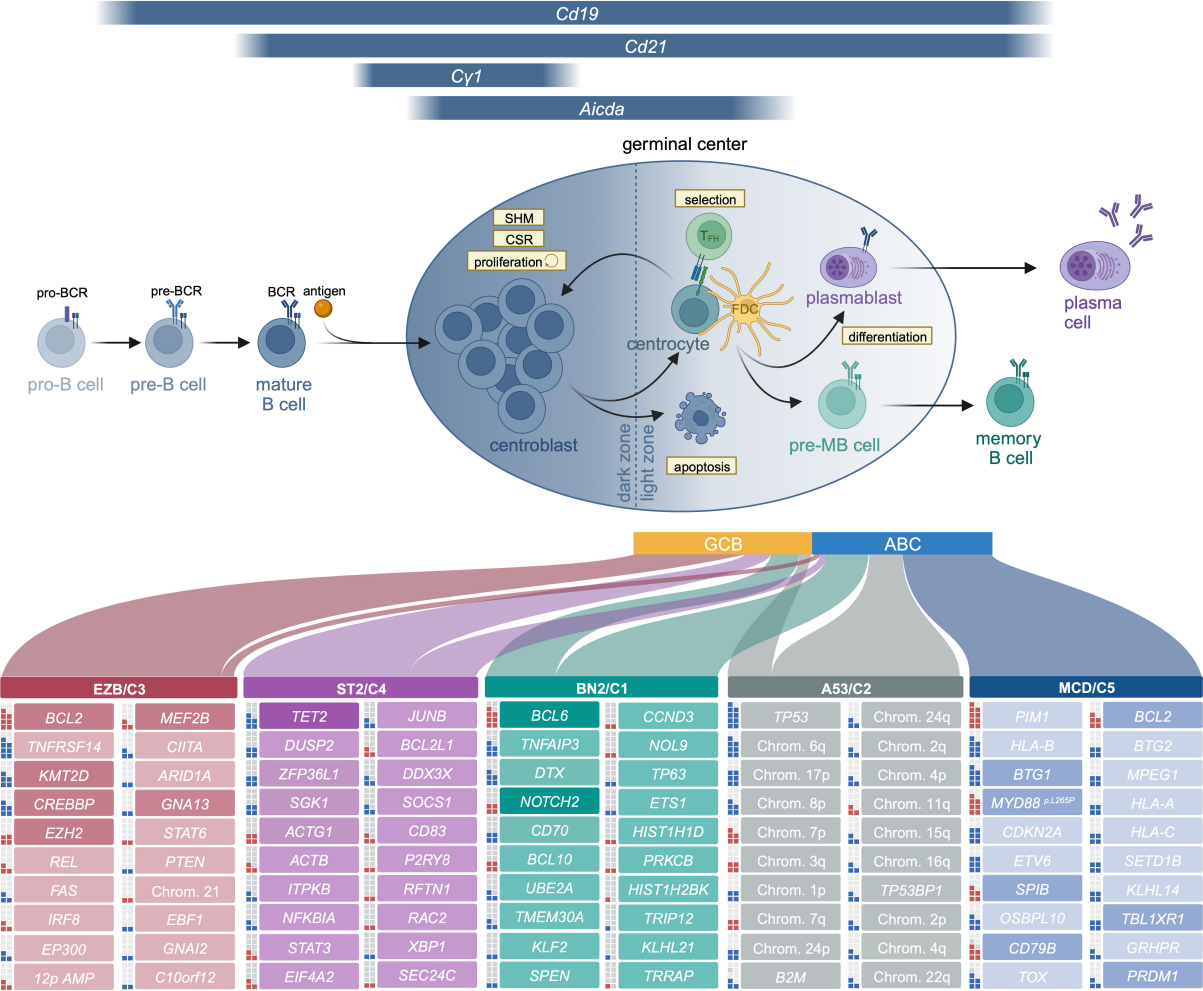
B cells develop in the bone marrow, where they pass through the pro- and pre-B cell stages before they enter the periphery as antigen-naïve mature B cells. Binding of antigen results in the activation of the B cell and its migration to the follicle, where the germinal center structure is formed. This structure is subdivided into two areas, the dark and the light zone. Proliferation predominantly occurs in the dark zone. Moreover, AID-driven somatic hypermutation (SHM), resulting in B cell receptor diversification, is also largely confined to the dark zone. B cells migrate to the light zone, where they compete for antigen presented by follicular dendritic cells (FDC) and CD40 stimulation provided by T follicular helper cells (T_FH_). Signals from T helper cells, either at the pre-GC stage or in the GC light zone promote class switch recombination (CSR). While high-affinity B cells are provided with those signals, prompting them to either recycle to the dark zone or differentiate into the memory B or plasma cell lineage, low-affinity B cells do not receive pro-survival stimuli and undergo apoptosis. While GCB DLBCL cells show features of light zone germinal center B cells, the ABC subtype more closely resembles plasmablast- or pre-MB stages. The frequency of ABC and GCB DLBCL within each genetic cluster is represented by the width of the respective link (unclassified cases are omitted for clarity of visualization but exist at different frequencies in all clusters: EZB, 9%; ST2, 22%; BN2, 42%; A53, 5%; MCD, 1%). The top 20 mutated genes for each genetic cluster are listed. Genes that have been investigated in a mouse model in the context of lymphoma are shaded with a higher color intensity. Squares to the left of each gene represent the frequency of alteration within the respective cluster (each square equals 10%, putative oncogenes are marked in red, tumor suppressors in blue). Frequencies of mutations, genetic clusters and transcriptomic subtypes are extracted from (28).

When generating mouse models of aggressive lymphoma, it is crucial to precisely phrase the question these models should address. Typically, the goal of generating mouse models is for these tools to serve as preclinical avatars of the human disease, which can be deployed to either understand the biology of certain genomic aberrations, or to serve as model systems for preclinical drug development. Particularly in the latter context, it is critical that the genomic context in which individual genetic aberrations are induced in the murine genome mimics the situation observed in human neoplastic lesions. In the subsequent paragraphs, we will discuss murine alleles with relevance to human lymphomagenesis and will describe allele combinations that led to the development of valuable preclinical models (**Figure 1** and **Table 1**). We will organize our discussion according to the clustering of mutations that occurs in human DLBCL (2, 3, 28).

**Table 1:** Overview of genes that are recurrently altered in human DLBCL.

Gene	Cluster	Allele name	Description	Reference
*BCL6*	C1/BN2	*IμHABcl6*	Knock-in of HA-tagged murine *Bcl6* cDNA into the *IgH* locus.	(44)
*NOTCH2*	C1/BN2	*Notch2IC*	Conditional expression of intracellular *Notch2* from the *Rosa26* locus.	(45)
*BCL2*	C3/EZB C5/MCD	*BCL2^Tracer^*	RAG-mediated activation of human *BCL2* expression.	(46)
		*VavP-BCL2*	Expression of human *BCL2* driven by the *Vav2* promoter.	(47)
		*R26^LSL.BCL2^*	Conditional overexpression of human *BCL2* from the *Rosa26* locus, co-expression of EGFP.	(48)
*ARGHEF1*	C3/EZB	*Arghef^null^*	Constitutive knockout of *Arghef1.*	(49, 50)
*CREBBP*	C3/EZB	*Crebbp^flox^*	Exon 9 of *Crebbp* is flanked by *loxP* sites.	(51, 52)
*EZH2*	C3/EZB	*Ezh2^Y641F^*	Conditional expression of *Ezh2^Y641F^* from the endogenous locus.	(53)
		*Ezh2^Y641F^*	Conditional expression of *Ezh2^Y641F^* from the endogenous locus.	(54)
		*Ezh2^Y641N^*	Conditional overexpression of *Ezh2^Y641N^* from the *Col1A* locus.	(55)
*FBXO11*	C3/EZB	*Fbxo11^flox^*	Exon 4 is flanked by *loxP* sites.	(56)
*GNA13*	C3/EZB	*Gna13^flox^*	Exons 1 and 4 of *Gna13* are flanked by *loxP* sites.	(49, 57)
*MEF2B*	C3/EZB	*Mef2b^stopD83V^*	Knock-in of a *loxP*-STOP-*loxP*-*Mef2b^D83V^* cassette into the endogenous locus.	(58)
*KMT2D*	C3/EZB	*Kmt2d^flox^*	Exons 16 to 19 of *Kmt2d* are flanked by *loxP* sites.	(59)
*S1PR2*	C3/EZB	*S1pr2^null^*	Constitutive knockout of *S1pr2.*	(49, 60)
*H1-2*	C4/ST2	*H1c^null^*	Constitutive knockout of *Histone 1* isoform c.	(61)
*H1-4*	C4/ST2	*H1e^null^*	Constitutive knockout of *Histone 1* isoform e.	(61)
*TET2*	C4/ST2	*Tet2^flox^*	Exon 3 of *Tet2* is flanked by *loxP* sites.	(62, 63)
*BTG1*	C5/MCD	*R26^LSL.BTG1.G36H^*	Conditional overexpression of *BTG1* ^p.G36H^ from the *Rosa26* locus.	(64)
*CD79B*	C5/MCD	*CD79b ^p.Y195H^*	Conditional expression of the *CD79b^p.Y195H^* mutation from the endogenous locus.	(65)
*MYD88*	C5/MCD	*Myd88^cond-p.L252P^*	Conditional expression of *Myd88* ^p.L252P^ from the endogenous locus.	(48)
		*Myd88^L252P^*	Conditional expression of *Myd88* ^p.L252P^ mutation from the endogenous locus, co-expression of *GFP*.	(66)
		*MYD88^L265P^*	Overexpression of human *MYD88* ^p.L265P^ from the *Col1A1* locus.	(67)
*PRDM1*	C5/MCD	*Prdm1^flox^*	Zinc finger motifs of *Prdm1* are flanked with *loxP* sites.	(68, 69)
*SPIB*	C5/MCD	*R26^LSL.Spib^*	Conditional overexpression of murine *Spib* from the *Rosa26* locus, co-expression of EGFP.	(68)
*TBL1XR1*	C5/MCD	*Tbl1xr1^p.D370Y^*	Conditional expression of the *Tbl1xr1* ^p.D370Y^ mutation from the endogenous locus.	(70)
		*Tbl1xr1^flox^*	Exon 5 of *Tbl1xr1* is flanked by *loxP* sites.	(70)

For each gene, the corresponding molecular cluster, as well as the respective murine allele together with a brief description and the specific reference are provided.

## Cluster 1/BN2 modeling

Structural variants involving *BCL6* are the hallmark feature of C1/BN2 DLBCL (2, 3). BCL6 itself is considered the master regulator of the GC reaction, and its continued expression prevents GC exit and terminal differentiation (11, 44, 71–73). *BCL6* expression is tightly controlled through various regulatory elements in the *BCL6* promoter region, including an IRF4 binding site. IRF4, in turn, acts as a transcriptional repressor involved in silencing *BCL6* expression to promote GC exit (19). In lymphoma, BCL6 expression is sustained through different mechanisms. These include indirect effects, such as mutational inactivation of *FBXO11* (56, 74), which is involved in mediating proteasomal degradation of BCL6, as well as mutations in *CREBBP* and *MEF2B*, which lead to enhanced and maintained *BCL6* transcription (75, 76). Additionally, genetic alterations directly affecting the *BCL6* gene, either though mutations within the 5’ non-coding region or via rearrangements and subsequent promoter substitution (19, 77–80). Collectively, these aberrations prevent GC exit by maintaining the BCL6-driven GC program, ultimately retaining B cells in a hyperproliferative state. In this state, BCL6-mediated blunting of the DNA damage response, for instance through repression of *TP53*, *ATR*, *CHK1*, *CDKN1A*, and others, facilitates genomic instability (4, 81–88).

In an attempt to model the human DLBCL- and FL-associated t (3, 14) (q27;q32) rearrangement, a knockin model was generated, where HA-tagged *Bcl6* was targeted into the murine *Igh* locus, placing it under the control of the *Iµ* promoter (44). These *Iµ^HA.Bcl6/wt^* animals displayed spontaneous GC hyperplasia, with a notable predominance of dark zone over light zone GC B cells. Moreover, these mice developed spontaneous lymphomas, albeit with a long latency of >15 months (44). These lesions closely resembled central aspects of human DLBCL with respect to morphology, presence of AID-mediated somatic hypermutation, as well as non-random clonal numerical and structural cytogenetic abnormalities. These abnormalities included *Myc-Igh* rearrangements, in the vast majority of cases (44). It is worth mentioning that conditional alleles of a number of genes that are frequently altered in *BCL6*-rearranged human DLBCL, such as *Tnfaip3*, *Notch2* and *Spen*, are readily available. Exploring the possibility of creating compound mutant models to investigate a potential oncogenic cooperation between these genes could be of great interest (3, 45, 89, 90).

## Cluster 2/A53 modeling

Cluster 2 DLBCLs are characterized by the prevalence of bi-allelic *TP53*-inactivating mutations and 17p copy number losses, which frequently co-occur with copy number losses affecting 9p21.13/*CDKN2A* and 13q14.2/*RB1*, as well as 1q23.3/*MCL1* copy number gains (3). Up to this point, no systematic cluster 2 DLBCL modeling has been reported. However, there are various *Trp53* alleles, including conditional knockout alleles, as well as alleles that permit the conditional expression of mutant versions of *Trp53* (91–93). Similarly, conditional *Rb1* knockout alleles and conditional *Mcl1* overexpression alleles are available, which would in principle allow cluster 2 DLBCL modeling attempts (94–97).

## Cluster 3/EZB DLBCL modeling

In human DLBCL, cluster 3/EZB cases are dominated by GCB-DLBCL cases with respect to cell of origin classification (3). Molecularly, cluster 3 DLBCL is dominated by co-occurring *BCL2* mutations and rearrangements that position *BCL2* downstream of the *IGH* enhancer (3). In addition to these *BCL2* aberrations, cluster 3/EZB harbors recurrent mutations in genes encoding the epigenetic regulators KMT2D, EZH2 and CREBBP, the transcription factor MEF2B and the Guanine Nucleotide-Binding Protein Subunit Alpha-13 (GNA13), as well as the F-box protein 11 (FBXO11), among others (2, 3, 28). Numerous alleles capturing these genes have been generated and we will discuss them in the following paragraphs.

The t(14;18) translocation can be detected in 85% of follicular lymphoma (FL) and 30% of GCB-DLBCL. This rearrangement, which places *BCL2* under the transcriptional control of the *IGH* regulatory elements, occurs early during B cell development, as a result of erroneous VDJ recombination. Intriguingly, this translocation, which clearly represents a selected event during B cell transformation, by itself does not have substantial transformative capacity, as the t(14;18) is detectable in approximately 70% of healthy adults who never develop FL or GCB-DLBCL. Several distinct alleles have been generated to model the t(14;18) translocation *in vivo* (46, 47, 98–100). For the purpose of this review, we will focus on the three most commonly used alleles in B-NHL modeling.

The *VavP-BCL2* allele was generated by juxtaposing the human *BCL2* cDNA to the *Vav2* promoter, which drives pan-hematopoietic expression of *BCL2* at a developmental stage earlier than the time at which the t(14;18) translocation typically occurs (47, 101). Despite the non-B cell-restricted *BCL2* expression in the *VavP-BCL2* model, these mice develop clonal, somatically *Ighv*-mutated B cell lymphomas, which display a follicular growth pattern, as well as PNA and BCL6 expression and lack of post-GC markers (47). *VavP-BCL2* mice were crossed with a number of relevant alleles, including *Crebbp^fl^*, *Kmt2d^fl^*, *Ezh2^Y641N^* and *Ezh2^Y641F^* to model aggressive B-NHL *in vivo* (see details below) (51, 102, 103). In addition, *VavP-BCL2*-derived hematopoietic stem cells were used as a platform to assess the effects of additional gain and loss of function genetic aberrations introduced by viral transduction prior to transplantation into irradiated recipient animals (55).

One of the major drawbacks of the *VavP-BCL2* model is the ubiquitous *BCL2* expression in the hematopoietic lineage. This limitation is circumvented by the introduction of the *BCL2-Ig* model, which was generated by classical transgenesis and expresses a *BCL2* minigene under the transcriptional control of *IG* regulatory elements (98). This strategy limits ectopic *BCL2* expression to the B cell lineage. When these transgenic animals were immunized with sheep red blood cells, they developed follicular lymphoma (40%) and plasmablastic lymphoma (20%) with a latency of more than 500 days (58).

The development of the mosaic *BCL2^Tracer^* transgenic model, in which RAG-mediated VDJ recombination flips exon 3 of a human *BCL2* sequence from inverse into direct orientation allowing correct splicing and subsequent expression of *BCL2* under the transcriptional control of a constitutive CMV promoter and 3’-located *IGH* intronic enhancer (Eµ), constitutes a further step in modeling the t(14; 18) (46). This design offers two critical advantages, as it faithfully mimics the sporadic nature of the t(14; 18) rearrangement, as well as the RAG recombinase-mediated occurrence of the recombination event at the pro-/pre- B cell developmental stage (46). It is important to note, however, that the *BCL2^Tracer^* mice did not develop spontaneous lymphomas during the reported 9 months observation period (46).

Next to *BCL2* aberrations, cluster 3 DLBCLs are characterized by recurrent mutations in the epigenetic modifiers KMT2D, EZH2 and CREBBP. KMT2D constitutes the catalytic activity of the complex of proteins associated with Set1 (COMPASS), which regulates transcriptional activity through mediating mono- and di-methylation of histone 3 lysine 4 (H3K4) at enhancer and super-enhancer regions within the chromatin. In human DLBCL and FL, *KMT2D* aberrations are typically loss of function events, either leading to truncations or deleterious missense mutations within the SET domain, indicating that *KMT2D* acts as a tumor suppressor gene. A *Kmt2d^fl^* allele is available (59) and when *Kmt2d* was deleted during early B cell development by *Cd19^Cre^*, the majority of *Cd19^Cre/wt^;Kmt2d^fl/fl^* mice died from lymphoma within 338 days. The developing oligoclonal lymphomas were of pre-GC origin and had undergone VDJ recombination. However, these lymphomas displayed no signs of class switch recombination or somatic hypermutation and lacked expression of the murine GC B cell marker PNA (104). Interestingly, when crossed with *Cγ1^Cre^*, *Kmt2d^fl^* animals did not develop lymphoma within an observation period of 18 months (103). However, *VavP-BCL2;Cγ1^Cre/wt^*;*Kmt2d^fl/fl^* mice developed significantly more clonal B cell lymphomas than *VavP-BCL2* controls (103). Morphologically, these lymphomas covered the entire spectrum from low to high-grade FL and DLBCL (103). These clonal lesions stained positive for B220, PAX5 and BCL6, displayed evidence of somatic hypermutation and were thus likely GC-experienced (103). These data indicate that GC-specific loss of *Kmt2d* cooperates with *BCL2* in GC B cell-derived FL and DLBCL lymphomagenesis.

As loss of *KMT2D* biochemically results in decreased (H3K4) methylation and subsequently altered gene expression, it was proposed that pharmacological inhibition of the KDM5 family, which mediates H3K4me3/me2 demethylation, might, at least partially, restore H3K4 methylation in *KMT2D*-deficient cells, leading to the reinstated expression of KMT2D-controlled genes. Indeed, KDM5 inhibition using a series of α-ketoglutarate-competitive small molecule inhibitors, promoted increased H3K4me3 levels and had a growth-repressing effect in *KMT2D*-defective cell lines and xenograft lymphoma models (105). Thus, KDM5 inhibition might be a viable therapeutic strategy for the treatment of KMT2D-mutant GC-derived lymphomas. However, high intracellular α-ketoglutarate concentrations complicate the use of α-ketoglutarate-competitive small molecule inhibitors. Hence, the development of potent KDM5 PROTAC degraders is highly desirable to further develop the concept of KDM5 repression for the treatment of *KMT2D*-mutant lymphomas.

The methyltransferase EZH2 is part of the Polycomb Repressive Complex 2 (PRC2) and catalyzes the deposition of repressive H3K27me3 marks at cell type and context-specific chromatin regions, which in GC B cells include genes that control proliferation and cell checkpoint signaling, including *Cdkn1a* and *Cdkn1b*, as well as genes involved in driving terminal plasma cell differentiation, such as *Prdm1* and *Irf4* (55, 106, 107). Approximately 30% of human GCB-DLBCLs and particularly cluster 3/EZB cases harbor heterozygous *EZH2* mutations, typically located within the EZH2 SET domain coding region affecting p.Y641 (2, 3, 28). These SET domain mutations appear to convey an enhanced trimethylation efficiency to mutant EZH2 (108).

Several *Ezh2* alleles have been generated and their role in B cell biology and lymphomagenesis has been investigated exhaustively. Important evidence for a critical role of *Ezh2* in GC B cell biology and GC formation was provided by the assessment of *Cγ1^Cre^;Ezh2^fl/fl^* mice, which displayed massively impaired GC B cell expansion following sheep red blood cell (SRBC) immunization-mediated GC induction. A similar lack of GC B cell expansion upon SRBC immunization was observed when C57BL/6 wildtype animals were treated with the EZH2 inhibitor GSK-503, firmly establishing a central role for EZH2 in establishing GCs (55).

To gain mechanistic insight into the biological function of the *EZH2* hotspot mutations observed in human DLBCL, two additional alleles were generated, namely an *Ezh2^Y641F^* and *Ezh2^Y641N^* strain. Both alleles are conditionally Cre-inducible. Expression of the *Ezh2^Y641F^* allele is driven by the endogenous *Ezh2* promoter and expression of the *Ezh2^Y641N^* allele is driven off a CAG promoter (53, 55). When crossed with *Cγ1^Cre^*, these animals did not develop overt lymphoma. However, further analyses revealed that following immunization, both *Cγ1^Cre^*;*Ezh2^Y641F/wt^* and *Cγ1^Cre^*;*Ezh2^Y641N/wt^* mice displayed GC hyperplasia and enhanced H3K27 trimethylation and subsequent silencing of EZH2 target genes, which appears to be dependent on BCL6 to ultimately form the CBX8- BCOR repressive complex (53, 55). Of note, reminiscent of the conditional *Kmt2d* knockout mice, when *Ezh2^Y641F^* mice were crossed with *Cd19^Cre^* animals in which recombination occurs at early B cell developmental stages, the resulting *Cd19^Cre/wt^*;*Ezh2^Y641F/wt^* mice develop DLBCL within 12 months (54). Lymphoma development in this setting was further enhanced when *Trp53* was co-deleted in *Cd19^Cre/wt^*;*Ezh2^Y641F/wt^*;*Trp53^fl/fl^* mice (54).

Next to an oncogenic cooperation between *Ezh2* and *Bcl6* (53), HSC transplant experiments revealed an oncogenic cooperation between *BCL2* and *EZH2 ^Y641N/F^* ^81,83^. In brief, *VavP-BCL2* HSCs were transduced with either empty vector, *Ezh2^wt^* or *Ezh2^Y641F^* constructs and subsequently transplanted into lethally irradiated recipient mice. These recipients were then immunized with SRBCs (monthly). Whereas the majority of *VavP-BCL2;Ezh2^Y641F^* and 20% of *VavP-BCL2;Ezh2^wt^* bone marrow chimeras developed lymphoma displaying morphological features of DLBCL with hepatosplenomegaly on day 111, none of the *VavP-BCL2* chimeras displayed overt lymphoma manifestation at that stage (55). Of note, results obtained in an autochthonous setting do not support these observations made in the transplant setting. Here, *Cγ1^Cre^*;*VavP-BCL2;Ezh2^Y641F/wt^* and *Cγ1^Cre^*;*VavP-BCL2;Ezh2^Y641N/wt^* mice did not succumb to lymphoma significantly earlier than *VavP-BCL2* controls (102). *VavP-BCL2*, *Cγ1^Cre^*;*VavP-BCL2;Ezh2^Y641F/wt^* and *Cγ1^Cre^*;*VavP-BCL2;Ezh2^Y641N/wt^* did, however, die significantly earlier than *Cγ1^Cre^*, *Cγ1^Cre^*;*Ezh2^Y641F/wt^* and *Cγ1^Cre^*;*Ezh2^Y641N/wt^* controls (102). It is important to note that these results are likely confounded by the development of glomerulonephritis and other autoimmune diseases which occur in *VavP-BCL2* mice with almost 100% penetrance, resulting in a median survival of around 6 to 9 months, representing a limiting factor for employing this allele in the study of GC B cell lymphomagenesis (47, 55, 61, 64, 109).

Next to the above-described role of *EZH2* gain-of-function mutations in B-NHL lymphomagenesis and chromatin remodeling, EZH2 also emerges as a potential drug target. By using the above-mentioned HSC transplant experiments, as well as the autochthonous *Cγ1^Cre^*;*VavP-BCL2;Ezh2^Y641F/wt^* and *Cγ1^Cre^*;*VavP-BCL2;Ezh2^Y641N/wt^* models, it was shown that expression of the MHCI and MHCII antigen presentation machinery was substantially reduced in *Ezh2*-mutant lymphoma cells, compared to wildtype controls (102). Coincidingly, *Ezh2*-mutant lymphomas were characterized by a drastically reduced T cell infiltration of the local lymphoma microenvironment (102). These observations suggest that mutationally enhanced EZH2 H3K27me3 activity promotes escape from lymphoma-suppressing immune surveillance through the repression of lymphoma cell autonomous MHCI/II expression. From a therapeutic point of view, it is interesting to note that pharmacological inhibition of mutant EZH2 activity with EPZ-6438 reduced H3K27me3 in DLBCL cell lines (102). Moreover, EPZ-6438 exposure significantly increased surface MHCI/II surface expression in the majority of the investigated *EZH2*-mutant GCB-DLBCL cell lines, whereas EZH2 wildtype cell lines did not display substantial changes in MHCI/II expression (102).

The KAT3 family histone and non-histone acetyl-transferase CREBBP is frequently affected in FL and DLBCL, either by truncating or missense mutations affecting the catalytic HAT domain (2, 3, 28, 75). In DLBCL, *CREBBP* mutations are enriched in C3 DLBCL (2, 3, 28). Biochemically, CREBBP, together with its paralog EP300, exerts transcriptional control through H3K18 and H3K27 enhancer and promoter acetylation. In a series of experiments using human lymphoma cell lines, RNAi-mediated depletion of *Crebbp* on a *VavP-BCL2* background and GC-specific conditional *Crebbp* knockout, two independent groups recently demonstrated that CREBBP loss promotes the development of GC-derived lymphomas *in vivo* (51, 109). Mechanistically, CREBBP loss-of-function led to substantially reduced H3K27 acetylation at enhancers and super-enhancers, leading to subsequent transcriptional repression of a series of genes involved in the BCR-, TLR- and CD40 receptor signaling cascades, genome maintenance and cell cycle regulation, as well as genes regulating GC and plasma cell development and antigen presentation, including the MHCII complex (51, 109). A further inspection of the affected genes revealed that CREBBP-regulated enhancers are largely overlapping targets of the BCL6/SMRT/HDAC3 complexes (109). The role of HDAC3 was confirmed using GC B cells derived from *Hdac3*-deficient mice (109). In these experiments, *Hdac3* deficiency rescued repression of the *Crebbp/BCL6*-regulated transcripts and suppressed *Crebbp*-mutant lymphomas *in vitro* and *in vivo* (109). Particularly this HDAC3 involvement is of potential therapeutic interest, as subtype-specific HDAC inhibitors are currently being developed and could potentially be used to treat *CREBBP*-mutant lymphomas (110–113). From a modeling point of view, it is important to note that homo- or heterozygous *Crebbp*-deficiency (in *Cd19^Cre/wt^;Crebbp^fl/fl^* or *Cd19^Cre/wt^;Crebbp^fl/wt^* and *Cγ1^Cre^;Crebbp^fl/fl^* or *Cγ1^Cre^;Crebbp^fl/wt^* mice) did not lead to significant lymphoma (FL/DLBCL) development (51). However, *Cγ1^Cre^;Crebbp^fl/wt^*;*VavP-BCL2* animals displayed a significant increase in lymphoma incidence, compared to *Cγ1^Cre^;Crebbp^fl/wt^* controls (51). These lesions mimicked critical aspects of human FL, including clonal GC B cell origin indicated by BCL6 expression, as well as lack of IRF4 and CD138 positivity and the presence of clonal *Ig* rearrangements carrying mutations as evidence of somatic hypermutation (51). There were no significant survival differences between *Cγ1^Cre^;Crebbp^fl/wt^*;*VavP-BCL2* and *Cγ1^Cre^;Crebbp^wt/wt^*;*VavP-BCL2* animals, which is likely due to the development of glomerulonephritis and other autoimmune diseases that develop in *VavP-BCL2* mice with almost 100% penetrance (47).

Next to genomic aberrations affecting epigenetic modifiers, such as EZH2, KMT2D, and CREBBP, transcription in is also rewired in GC-derived lymphomas via mutations in the gene encoding the transcription factor MEF2B. In B cells, *MEF2B* expression is restricted to GC B cells, where it controls the expression of a plethora of genes, including *BCL6* (58, 76). Approx. 15% of DLBCL cases display protein-damaging *MEF2B* mutations (2, 3, 28), affecting either the N-terminal DNA-binding domain or the C-terminal protein tails, which has been shown to be the target of different post-transcriptional modifications, including phosphorylation and sumoylation (5, 76). Particularly the effect of the MEF2B p.D83V mutation was investigated in more detail (58). These experiments revealed that MEF2B^wt^-driven transcription of a luciferase reporter was readily abrogated when either HDAC5, HDAC7, or CABIN1 (which is part of the HUCA complex consisting of HIRA, UBN1, CABIN1, and ASF1A) were co-expressed (58). In marked contrast, MEF2B^D83V^-mediated reporter expression was unaffected by HDAC5, HDAC7, or CABIN1 co-expression, indicating that this mutation mediates escape from negative regulation (58). These data were further corroborated through the generation of a Cre-inducible *Mef2b^D83V^* allele in which a loxP-STOP-loxP (LSL) cassette was inserted into the endogenous locus followed by an exon 3 harboring the p.D83V mutation (58). When these animals were crossed with *Cd21^Cre^*, the resulting *Cd21^Cre/wt^*;*Mef2b^D83V/wt^* mice displayed an increased abundance of GC B cells, as well as GC hyperplasia, compared to *Mef2b* wildtype controls (58). Moreover, approximately 20% of *Cd21^Cre/wt^*;*Mef2b^D83V/wt^* mice developed lymphomas (FL and DLBCL) at late time points. In a further extension of these experiments, *Cd21^Cre/wt^*;*Mef2b^D83V/wt^*;*BCL2-Ig* mice were generated (58). More than 90% of these animals developed GC-experienced, somatically hypermutated clonal lymphomas, in which the malignant lesions morphologically and phenotypically mimicked human FL and DLBCL (58).

While C3 DLBCL is clearly enriched for mutations in genes encoding epigenetic modifiers, transcription factors and *BCL2*, two additional genes with different functions are also recurrently altered in C3 DLBCL and will be discussed briefly. These are *GNA13* and *FBXO11*. The guanine nucleotide binding protein Gα13 (encoded by *GNA13*) serves as a signaling molecule downstream of the G-protein-coupled receptor sphingosine-1 phosphatase receptor-2 (S1PR2) and relays signals mediating physical confinement of B cells in the GC through repression of migration and negative regulation of the AKT pathway (5, 14, 114). The Gα13 pathway is affected by inactivating mutations in approximately 20% of GCB-DLBCL, including lesions in *GNA13*, *S1PR2*, *ARHGEF1* and *P2RY8* (2, 3, 5, 14, 49). Moreover, *S1PR2* was recently shown to act as a tumor suppressor in ABC-DLBCL and *S1PR2* expression was prognostic in that setting (115). The biology of Gα13 in lymphoma was recently further dissected through the generation of two distinct B cell-specific loss-of-function models, namely a GC B cell-specific deletion in the *Aicda^Cre^*;*Gna13^fl/fl^* setting (116) and a pan-B cell disruption of Gα13 expression in the *Mb1^Cre/wt^*;*Gna13^fl/fl^* setting (49). In both scenarios, animals displayed an increased abundance of GC B cells, disrupted GC anatomy with defective spatial distribution of dark zone and light zone B cells, irregular B cell migratory phenotypes and an increased somatic hypermutation activity. It is important to note that *S1pr2* deficiency does not fully phenocopy *Gna13* loss. While *Gna13*- and *Arhgef1* deficiency lead to leukemic effusion of GC B cells into the lymph- and blood stream, *S1pr2*-deficiency does not (49). This incomplete phenocopy fueled the hypothesis that additional Gα13-coupled G-protein-coupled receptors might regulate GC confinement. Indeed, in human DLBCL and Burkitt’s lymphoma sequencing data, the orphan receptor P2RY8 was identified as recurrently mutated. And while there is no murine orthologue of human *P2RY8*, overexpression of *P2RY8* repressed GC B cell proliferation in murine Peyer’s patches and mesenteric lymph nodes, reminiscent of what could be observed with *S1pr2* overexpression. Importantly, *P2RY8*-mediated GC growth suppression required the presence of Gα13 and was undetectable in *Gna13*-deficient settings, indicating that multiple G-protein-coupled receptors signal via Gα13 to confine GC B cells to the GC. Moreover, *S1pr2* constitutive knockout mice spontaneously develop clonal GC-derived DLBCL-like lymphomas displaying increased AID activity with approximately 50% penetrance, lending further support to the hypothesis that the Gα13 pathway is critically involved in lymphomagenesis (117).

FBXO11 belongs to the family of F-box proteins (118). The F-box is a protein domain consisting of approximately 40 amino acids, which primarily functions as a protein:protein interaction domain (118). It is through this F-box domain that F-box proteins interact with other components of SCF ubiquitin ligase complexes, which consist of SKP1, CUL1 and an F-box protein, such as FBXO11 (118). SCF ubiquitin ligase complexes deploy F-box proteins for the purpose of specific substrate recognition to ultimately drive the proteasomal degradation of a selected set of target proteins (119). F-box proteins are further subdivided according to the presence of additional protein domains into an FBXW family (harboring a WD40 domain), and FBXL family (containing a Leucine-rich repeat) and an FBXO family (F-box only or F-box and other domains) (119). It is through these non-F-box domains that F-box proteins confer substrate specificity to the SCF complex (119).

FBXO11-containing SCF complexes mediate the ubiquitinylation and subsequent proteasomal degradation of a number of lymphomagenesis-relevant substrates, including PRDM1 and BCL6 (5, 74, 119, 120). *FBXO11* is affected by deleterious genomic aberrations in approximately 5% of human DLBCL cases, particularly in C3 DLBCL and these *FBXO11* aberrations are associated with increased *BCL6* expression levels (2, 3, 28, 74). The *in vivo* effects of *Fbxo11* deletion in GC B cells was assessed in *Cγ1^Cre/wt^*;*Fbxo11^fl/fl^* and *Cγ1^Cre/wt^*;*Fbxo11^fl/wt^* mice, in which exon 4 of the *Fbxo11* gene was flanked by loxP sites (56). Analyses in these animals revealed that upon immunization *Fbxo11* deficiency in GC B cells leads to an increased number of GC B cells, GC hyperplasia, a shift within the GC B cell compartment towards an increased percentage of dark zone cells, compared to light zone B cells, as well as increased *BCL6* expression as a function of *Fbxo11* gene dose (56). The role of *Fbxo11* as a tumor suppressor in lymphomagenesis was formally established in *Cγ1^Cre/wt^*;*Fbxo11^fl/fl^* and *Cγ1^Cre/wt^*;*Fbxo11^fl/wt^* mice that received 6 immunizations with SRBCs over the course of their lifespan (56). In these experiments, animals were euthanized at 17 to 18 months. At autopsy, 5% of wildtype controls, approximately 35% of *Cγ1^Cre/wt^*;*Fbxo11^fl/wt^* mice and approximately 20% of *Cγ1^Cre/wt^*;*Fbxo11^fl/fl^* animals developed various types of lymphoproliferation, including florid follicular hyperplasia, lymphoproliferative disease and DLBCL (56). Particularly in the *Cγ1^Cre/wt^*;*Fbxo11^fl/wt^* setting, DLBCL development was recorded (56).

While the above-detailed models cover a lot of ground in DLBCL modeling, it is important to note that a recent comprehensive genomic analysis revealed that human DLBCLs harbor a median of 17 (range 0-48) genetic drivers (3). A further analysis provided an additional dissection of human EZB cases into those that displayed the so-called “DHIT gene expression signature (DHIT^+^)” and those lacking this signature (28). The DHIT signature is derived from double hit lymphomas, which harbor combined structural variants affecting *MYC* and *BCL2* (28). In this dataset, DHIT^+^ cases displayed a significantly worse overall survival, than DHIT^-^ cases (28). Moreover, a significant co-clustering of *MYC* (46%) and *TP53* (43%) aberrations was observed within the DHIT^+^ cases (28). In contrast, *MYC* and *TP53* lesions were observed in DHIT^-^ cases in only 4% and 15%, respectively (28). Mutations in other genes, such as *GNA13*, *DDX3X* and *FOXO1* were also enriched in the DHIT^+^ cases, albeit less pronounced (28). These observations provide a framework for the design and generation of more advanced C3/EZB models, such as *Cγ1^Cre/wt^*;*Ezh2^Y641F/wt^*;*Rosa26^LSL.Bcl2/LSL.Myc^*;*Tp53^fl/fl^*.

## Cluster 4/ST2 modelling

Cluster 4 DLBCL is dominated by highly recurrent mutations in genes encoding for the histone H1 isoforms H1B, C, D, and E. We note, however, that these *H1* mutations do not exclusively occur in C4 DLBCL, but can also be detected in other clusters, such as the C5/MCD cluster. Mechanistically, these so-called linker-histones bind to nucleosomes to promote chromatin compaction, which essentially enables the folding of chromatin into higher-order structures, and subsequently allowing the further epigenetic regulation through the recruitment of additional histone modifiers (121). Lymphoma-associated histone H1 mutations, most commonly affecting *H1C* and *H1E* are typically missense mutations within the globular C-terminal domain, which impairs chromatin binding (61, 122, 123). The tumor-suppressive role of *H1c* and *H1e* was recently confirmed *in vivo*, using constitutive *H1c^-/-^*;*H1e^-/-^* animals (61). Deploying chromatin conformation capture analyses in sorted *H1c^-/-^*;*H1e^-/-^* and wildtype control GC B cells revealed a profound architectural remodeling of the genome characterized by numerous focal shifts in chromatin constitution from a compacted to a relaxed state (61). In these cells, chromatin decompaction was associated with increased histone H3K36 dimethylation, as well as reduced H3K27 trimethylation (61). A further analysis demonstrated that decompacted genes in *H1c^-/-^*;*H1e^-/-^* GC-B cells were enriched for iPS cell reprogramming, mesenchymal-transition states, stem cell transcription factor cistromes, as well as H2K27me3-marked genes in haematopoietic cells (61). In subsequent competitive bone marrow chimera experiments, it was shown that *H1c^-/-^*;*H1e^-/-^* GC-B cells displayed a competitive advantage within the GC, which was associated with an increased abundance of cycling light zone B cells (61). Fittingly, *H1c^-/-^*;*H1e^-/-^*;*VavP-BCL2* animals showed a prominent disruption of lymph node architecture and infiltration of extranodal tissues, such as lung and liver with immunoblastic cells together with a prominent CD3-positive T cell infiltrate (61). Survival monitoring of different cohorts of experimental mice revealed that *H1c^wt/-^*;*H1e^wt/-^*;*VavP-BCL2* and *H1c^-/-^*;*H1e^-/-^*;*VavP-BCL2* animals displayed a significantly shorter overall survival than *VavP-Bcl2* controls (61). In these experiments, a trend towards increased lethality of *H1c^wt/-^*;*H1e^wt/-^*;*VavP-BCL2*, compared to *H1c^-/-^*;*H1e^-/-^*;*VavP-BCL2* animals was observed, possibly indicating that certain isoform specific functions, such as interactions with additional epigenetic modifiers, need to be retained for a full-blown competitive advantage of *H1c* and *H1e*-defective GC-B cells (61). This observation is also in line with the observation that *H1* mutations typically occur in a heterozygous fashion in human DLBCL (2, 3, 61).

*TET2* (ten-eleven translocation 2), the gene encoding the methylcytosine dioxygenase 2, which catalyzes the conversion of the modified genomic base 5-methylcytosine (5mC) into 5-hydroxymethylcytosine (5hmC), is mutated in approximately 5-10% of human GCB-DLBCL. *TET2* mutations are enriched in ST2 cluster DLBCL, which shares genomic features with C4 DLBCL (2, 3, 28, 124). *TET2* mutations are associated with clonal hematopoiesis and consistently patients with *TET2*-mutant lymphoma typically carry the identical mutation in their hematopoietic stem cells (HSCs) (125). Whether these *TET2* aberrations play a causal role in B cell lymphomagenesis in these patients or merely represent a clonal hematopoiesis scar was systematically assessed in experimental mice by conditional deletion in either HSCs (*Vav^Cre^*) or specifically in the B cell lineage (*Cd19^Cre^*) (62). In these models, *Tet2* deficiency led to a stalled transit GC transit of B cells and subsequent preneoplastic GC hyperplasia, reduced class switch recombination and a block in terminal plasma cell differentiation (62). Of note, *Tet2* disruption at the GC stage in *Cg1^Cre/wt^*;*Tet2^fl/fl^* mice did not lead to an expansion of GC B cells or any other detectable shift in the relative abundance of different B cell populations, compared to *Cg1^Cre/wt^*;*Tet2^wt/wt^* animals (62). Consistent with the impaired GC exit phenotype in *Cd19^Cre^*;*Tet2^fl/fl^* and *Vav^Cre^*;*Tet2^fl/fl^* mice, *Tet2* deficiency was associated with reduced enhancer cytosine hydroxymethylation and reduced expression of genes that promote GC exit, such as *Prdm1*, in these settings (62).* A* further analysis revealed that there is substantial overlap of the enhancers and genes that are repressed in *Tet2*-deficient settings and *CREBBP*-mutant lymphomas, suggesting a similarly rewired transcriptome that is brought about by these distinct genomic lesions (62). Perhaps not surprisingly, *TET2* and *CREBBP* mutations are mutually exclusive in human lymphomas (62).

## Cluster 5/MCD modeling

Cluster 5/MCD DLBCL is dominated by ABC-DLBCL cases and is enriched for co-occurring mutations leading to activation of BCR signaling (*CD79B*) and the Toll-like receptor (TLR) pathway (*MYD88*, particularly through the highly recurrent p.L265P hotspot mutation) (2, 3, 28, 48, 65, 68, 126, 127). In addition, C5/MCD DLBCL cases almost uniformly harbor copy number gains on 18q, which, among others, includes the *BCL2* locus (2, 3, 28). BCL2 gains and amplifications are a defining feature of MCD DLBCL (2, 28). C5/MCD cases are further enriched for aberrations leading to a block in plasma cell differentiation (*SPIB* gains and loss of function lesions affecting *PRDM1* and *TBL1XR1*), as well as lesions mediating escape from immune surveillance (*HLA-A*) (2, 3, 28, 68, 128).

A number of relevant alleles mimicking recurrent aberrations in C5/MCD DLBCL have been generated in recent years and have enabled a precise modelling of this DLBCL subtype (48, 64, 65, 68–70, 127). A first step towards generation of a C5/MCD model was the development of a conditional *Myd88^p.L252P^* allele (*Myd88^c-p.L252P^*) that is expressed from the endogenous locus upon Cre-mediated recombination (48). In this model, murine *Myd88^p.L252P^* is at the orthologous position of human *Myd88^p.L265P^* 116. When this *Myd88^p.L252P^* allele was crossed with *Cd19^Cre^*, *Aicda^Cre^* or *Cd21^Cre^* mice, all of the resulting animals developed splenomegaly with disrupted splenic architecture and non-clonal lymphoproliferative infiltration of the liver, as well as clonal DLBCL-like disease in a subset of cases (25%, 33% and 33%, respectively) (48).

In parallel, with the goal of mimicking *BCL2* amplification *in vivo*, a novel conditional allele in which *BCL2.IRES.GFP* was targeted into the *Rosa26* locus, was developed (48). This modeling strategy is rationalized by the observation that, in contrast to C3/EZB DLBCL where *BCL2* is typically affected by structural variants, *BCL2* is typically amplified in C5/MCD (2, 3, 28). In this model, expression of human *BCL2* cDNA from the *Rosa26* locus is prevented by the insertion of a *loxP.STOP.loxP* cassette upstream of the translation-initiating codon (48).

In a next step *Cd19^Cre/wt^*;*Myd88^p.L252P/wt^*;*Rosa26^LSL.BCL2.IRES.GFP/wt^* mice were generated, which expired significantly earlier than *Cd19^Cre/wt^*;*Myd88^p.L252P/wt^*, *Cd19^Cre/wt^*;*Rosa26^BCL2.IRES.GFP/wt^* and *Cd19^Cre/wt^* controls (48). *Cd19^Cre/wt^*;*Myd88^p.L252P/wt^*;*Rosa26^LSL.BCL2.IRES.GFP/wt^* animals almost uniformly succumbed to clonal lymphoma (penetrance of 83%), which displayed morphological features of DLBCL and stained positive for IRF4 and CD138, while being negative for B220 and BCL6, consistent with a plasmablastic, rather than a classical DLBCL phenotype (126).

A further refinement of the model was recently published, where the parental *Cd19^Cre/wt^*;*Myd88^p.L252P/wt^*;*Rosa26^LSL.BCL2.IRES.GFP/wt^* model was modified to either harbor a conditional *Prdm1* loss or conditional *Spib* overexpression from the *Rosa26* locus, in order to install a robust plasma cell differentiation blockade in the resulting *Cd19^Cre/wt^*;*Myd88^p.L252P/wt^*;*Rosa26^LSL.BCL2.IRES.GFP/wt^*;*Prdm1^fl/fl^* and *Cd19^Cre/wt^*;*Myd88^p.L252P/wt^*;*Rosa26^LSL.BCL2.IRES.GFP/LSL.Spib.IRES.GFP^* mice (68). As expected these mice developed DLBCL-like lymphomas and displayed a significantly reduced overall survival, compared to the *Cd19^Cre/wt^*;*Myd88^p.L252P/wt^*;*Rosa26^LSL.BCL2.IRES.GFP/wt^* parental strain (68). A more detailed molecular analysis using whole-exome sequencing, transcriptomics, flow-cytometry, as well as mass cytometry revealed that *Prdm1*- or *Spib*-altered lymphomas display molecular features of pre-memory and light-zone B cells, whereas lymphomas derived from the parental *Cd19^Cre/wt^*;*Myd88^p.L252P/wt^*;*Rosa26^LSL.BCL2.IRES.GFP/wt^* strain were enriched for late light-zone and plasmablast-associated molecular features (48, 68, 126). Thus, in contrast to the parental strain, which rather represents a model of plasmablastic lymphoma, engineering of a B cell-specific *Prdm1* deletion or *Spib* overexpression converts this model into a disease that faithfully resembles C5/MCD DLBCL (48, 68, 126).

In a further refinement of this model, a conditional allele that expresses the *Cd79b* p.Y195H mutation from the endogenous locus, mimicking the recurrent p.Y196H mutation within the *CD79B* immunoreceptor tyrosine-based activation motif (ITAM) found in human DLBCL cases, was generated (65). When four hallmark genetic aberrations in C5/MCD DLBCL were modelled in *Aicda^Cre/wt^*;*Myd88^p.L252P/wt^*;*Cd79b^p.Y195H/wt^*;*Rosa26^LSL.BCL2.IRES.GFP/wt^*;*Prdm1^fl/fl^* mice, these animals displayed a drastically increased in GC size and number at 8 weeks of age, compared to controls (65). *Aicda^Cre/wt^*;*Myd88^p.L252P/wt^*;*Cd79b^p.Y195H/wt^*;*Rosa26^LSL.BCL2.IRES.GFP/wt^*;*Prdm1^fl/fl^* animals developed splenomegaly and B cells in these animals displayed immune-phenotypes consistent with dark zone GC B cells and memory B cells (65). A further analysis, using bone marrow chimeras, revealed that 5 of 8 *Aicda^Cre/wt^*;*Myd88^p.L252P/wt^*;*Cd79b^p.Y195H/wt^*;*Rosa26^LSL.BCL2.IRES.GFP/wt^*;*Prdm1^fl/fl^* animals displayed disrupted splenic architecture due to a proliferation of large atypical lymphoid cells that morphologically resembled DLBCL (65). 2 of 5 animals carrying these intra-splenic lymphomas also harbored retroperitoneal lesions involving lymph nodes or accessory splenic tissue (65). Unfortunately, there was no further analysis of lymphomagenesis in the autochthonous setting, preventing a direct comparison with previous versions of C5/MCD modeling approaches. At first glance, the use of the *Cd19^Cre^* allele, which drives recombination already at the pro-B cell stage of B cell development, in the *Cd19^Cre/wt^*;*Myd88^p.L252P/wt^*;*Rosa26^LSL.BCL2.IRES.GFP/wt^*, *Cd19^Cre/wt^*;*Myd88^p.L252P/wt^*;*Rosa26^LSL.BCL2.IRES.GFP/wt^*;*Prdm1^fl/fl^* and *Cd19^Cre/wt^*;*Myd88^p.L252P/wt^*;*Rosa26^LSL.BCL2.IRES.GFP/LSL.Spib.IRES.GFP^* models could be perceived as not ideal. However, in this regard it is important to note that particularly the *MYD88* ^p.L265P^ mutation is detectable at low allele frequency in CD34-positive hematopoietic stem cells and B cell precursor cells of patients with lymphoplasmacytic lymphoma/Waldenstrom´s macroglobulinemia (127). These data indicate that the *MYD88* ^p.L265P^ mutation may arise early during B cell development and may require additional hits that are acquired during the GC reaction to facilitate full-blown transformation, similar to the t(14;18) rearrangements in FL, that are caused by the RAG recombinase, which is active in pre-B cells but not mature B cells. Moreover, the *Cd19^Cre/wt^*;*Myd88^p.L252P/wt^*;*Rosa26^LSL.BCL2.IRES.GFP/wt^*, *Cd19^Cre/wt^*;*Myd88^p.L252P/wt^*;*Rosa26^LSL.BCL2.IRES.GFP/wt^*;*Prdm1^fl/fl^* and *Cd19^Cre/wt^*;*Myd88^p.L252P/wt^*;*Rosa26^LSL.BCL2.IRES.GFP/LSL.Spib.IRES.GFP^* models display features of GC passage, including evidence of somatic hypermutation and class-switch recombination, which again suggests their GC origin (48, 68, 126). Against this background, a dual recombinase strategy enabling early *Myd88* mutation and GC-specific mutation of the additional C5/MCD hallmark genes (such as *Cd79b*, *Bcl2*, *Prdm1* and others) may be a desirable strategy for future modeling attempts.

An additional gene that is frequently affected by mutations in C5/MCD DLBCL is *TBL1XR1*, which encodes for a core component of the SMRT/NCOR1 complex, which in is recruited to chromatin by BCL6 in GC B cells (129). Biochemical experiments using a *TBL1XR1* p.Y446S mutant, which frequently occurs in human DLBCL, revealed that although the interaction with the SMRT/HDAC3 complex was preserved, the only significant change in the TBL1XR1^mut^ interactome was a robust shift away from an interaction with BCL6 towards an interaction with the transcription factor BACH2 (70), which plays a critical role in memory B cell generation (130). These data indicate that mutant *TBL1XR1* may redirect the SMRT complex toward BACH2, which in turn drives a transcriptional response mediating memory B cell fate in parallel to installing a plasma cell differentiation block via maintained BACH2-mediated *PRDM1* transcriptional repression (70).

To study the potential role of *Tbl1xr1* in lymphomagenesis, a *Tbl1xr1* allele, which allows conditional expression of the p.D370Y mutation from the endogenous locus, was generated (70). In line with transcriptional rewiring downstream of mutant *Tbl1xr1*, RNA-Seq analysis of GC B cells derived from these animals revealed an enrichment for ABC-DLBCL-associated gene expression signatures and NF-kB signaling, which are normally repressed in GC B by the BCL6-SMRT complex (70). Interestingly, it was also observed that GC B cells derived from *Tbl1xr1* p.D370Y mutant mice displayed de-repression of *Gpr183* and *S1pr1*, which are transcriptionally silenced in the wildtype setting (70). Of note, silencing of *Gpr183* and *S1pr1* is critical for containment of GC B cells in lymphoid follicles (70). Thus, de-repression of these genes may drive expansion of post-GC B cells, as well as extra-nodal accumulation of these cells. Further support for a tumor-suppressive role of *TBL1XR1* stems from the analysis of immunized *VavP-BCL2*;*Cd19^Cre/wt^*;*Tbl1xr1^fl/fl^* animals, which were shown to develop predominantly extra-nodal lymphomas, at a time point at which no tumors were detectable in *VavP-BCL2*;*Cd19^Cre/wt^* controls (70). B220+ cells in the *VavP-BCL2*;*Cd19^Cre/wt^*;*Tbl1xr1^fl/fl^* setting largely lacked GC B cell markers and displayed a relative expansion of (pre-) memory B cell populations (70). Morphologically, tumors in *VavP-BCL2*;*Cd19^Cre/wt^*;*Tbl1xr1^fl/fl^* mice were largely composed of large atypical immunoblasts, mimicking human extranodal ABC-DLBCL (70). These cells infiltrated extra-nodal tissues, such as liver and kidneys, while mostly sparing the lymph nodes (70). Tumors showed somatic mutations in the *J_H_4* intron and the *Pim1* locus (a known AID off-target), indicative of GC passage (70). Similar results were obtained in *VavP-BCL2*;*Cγ1^Cre/wt^*;*Tbl1xr1^fl/fl^* animals (70). Altogether, these data pinpoint a role for *TBL1XR1* in skewing the transcriptome towards a memory B cell phenotype and a plasma cell differentiation block, which may promote GC re-entry instead of plasma cell differentiation upon antigen recall.

While the selection of *TBL1XR1* mutations in C5/MCD DLBCL help to explain their memory B cell phenotypes and a plasma cell differentiation block, an explanation for the competitive fitness advantage of these cells remained largely elusive. A recent study focusing on the role of B cell translocation gene 1 (*BTG1*) helped to shed light on the mechanistic basis of competition within the GC reaction (64). *BTG1* mutations are detected in approximately 70% of C5/MCD DLBCL and are typically heterozygous missense aberrations, clustering at the N-terminal portion of the protein between an N-terminal hydrophobic domain and the LxxLL motif (2, 3, 28, 64). Particularly the glutamine residue in position 36 is most frequently replaced by a histidine (64). It was further shown that these N-terminal aberration can induce conformational changes of BTG1 (131). To assess the biological role of *BTG1* in the GC, two alleles were generated, namely a *Rosa26^LSL.BTG1.G36H^* and *Rosa26^LSL.BTG1.wt^* allele, where expression of either the mutant or the wildtype is prevented by a *loxP-STOP-loxP* cassette in the absence of Cre recombinase (64). The competitive fitness of *BTG1* p.Q36H-mutant B cells was assessed in an elegant *in vivo* experimental system, in which *Cd19^Cre/wt^*;*Rosa26^LSL.BTG1.G36H/wt^*;*Cd45.1* and *Cd19^wt/wt^*;*Rosa26^LSL.BTG1.G36H/wt^*;*Cd45.1* mice were crossed with a *B1-8^hi^* allele, which encodes a B cell receptor with high (4-hydroxy-3-nitrophenyl)acetyl (NP) antigen affinity in B cells with a Λ-immunoglobulin light chain that facilitates GC entry upon NP immunization (64, 132). Upon adoptive transfer of these cells into *Cd45.2* wildtype recipients, and subsequent immunization with the T cell-dependent antigen NP-ovalbumin, *BTG1* p.Q36H-mutant B cells displayed a progressive competitive advantage, reaching approximately 90% of all GC B cells at day 14 (64). In contrast, no competitive advantage was observed when the *Rosa26^LSL.BTG1.wt^* allele was used (64). Mechanistically, it was shown that *BTG1*-mutant B cells displayed a significant enrichment of gene set signatures associated with T_FH_ cell help, light zone to dark zone recycling B cells and MYC/mTORC1 signaling, compared to controls (64). Further experiments revealed that BTG1 represses *MYC* on a posttranscriptional level and that this repressive function is lost in mutant BTG1 (64). The oncogenic potential of the *BTG1* p.Q36H mutation was further investigated in a bone marrow chimera setting, where recipients transplanted with *Cγ1^Cre/wt^*;*VavP-BCL2*;*Rosa26^LSL.BTG1.G36H/wt^* HSCs developed clonal DLBCL-like lymphoma with evidence of somatic hypermutation and passed away significantly earlier than *Cγ1^wt/wt^*;*VavP-BCL2*;*Rosa26^LSL.BTG1.G36H/wt^* and *Cγ1^wt/wt^*;*Rosa26^LSL.BTG1.G36H/wt^* controls (64). Moreover, *Cγ1^Cre/wt^*;*VavP-BCL2*;*Rosa26^LSL.BTG1.G36H/wt^* transplanted animals displayed extra-nodal infiltration of malignant B cells into lungs, kidneys, and liver (64).

Next to the above-discussed genomic aberrations, C5/MCD lymphomas frequently display *CDKN2A* deletions, which through its gene products p16 and p19 controls the RB1 and TP53 pathways, respectively (2, 28). A number of conditional *Cdkn2a* alleles exist and it would be interesting to assess the role of *Cdkn2a* deletions on the background of the various C5/MCD models detailed above. It might be particularly interesting to assess whether *Cdkn2a* deficiency impacts lymphoma tropism and might promote primary CNS lymphoma development, which frequently harbors a C5/MCD genetic makeup (3, 133).

## Patient-derived mouse models of aggressive lymphoma

Human cancer cell line-derived xenograft (CDX) models are heavily used for *in vivo* pharmacology studies. The relatively low-cost and usability of CDX models make them attractive preclinical tools, however they typically do not fully recapitulate the disease complexity (134). CDX models are often cultured 2D over many passages in serum-containing media and implanted into immuno-compromised mice, such as NOD-SCID or Nude mice for drug efficacy studies (135). With the immune system as a crucial component of the antitumor response, and as immune-checkpoint-inhibitors (ICIs) are emerging as the standard of care for several cancer indications, this type of immuno-deficient mouse model system is not ideal for representing human biology (136). Furthermore, the abysmal correlation between therapeutic efficacy shown in CDX models and efficacy in humans, calls for innovation in preclinical mouse models, as <5% of clinical-stage cancer drugs reach regulatory approval (137).

A more complex preclinical mouse model system compared to CDX models are patient-derived xenograft models (PDX). PDX models are established from implanting tumor tissue from a patient into an immuno-compromised or humanized mouse. In contrast to CDX models, PDX models are not artificially grown and selected *in vitro* prior to implantation and are instead serially passaged in mice (138). One powerful advantage of PDX models is that the tumors can maintain the molecular heterogeneity of the patient sample. PDX models have been shown to exhibit clonal dynamics and acquired mutations, which emulate a similar trajectory as the primary tumor, and this is maintained over serial passages. This consistent clonal evolution between primary patient samples and PDX models give them a superior advantage over CDX models for functional analyses, and are ultimately better predictors of clinical response (139, 140). However, the PDX model system ultimately comes with its own limitations. First, commercially available PDX models are substantially more costly than CDX models. Establishing and maintaining an internal PDX biobank also comes with its financial, logistic and regulatory hurdles with regards to the acquisition of primary patient material and laboratory animal welfare (141). Another disadvantage of PDX models is the lack of the tumor microenvironment and human stromal component that is inherently missing in immuno-compromised mice. The main approach to bridge the human immune system and patient-derived tumors in mice is the humanized PDX model system. Humanized PDX models are developed by implanting human CD34+ cells from the umbilical cord or human PBMCs in irradiated immuno-deficient mice followed by the implantation of a patient-derived tumors. However, difficulties with the engraftment rate and the onset of graft-versus-host disease, due to HLA-mismatches between the donor and host are several roadblocks that have slowed the progress of this model system (142, 143).

Several PDX platforms of aggressive lymphomas have been established in an attempt to faithfully recapitulate the human disease biology and enable preclinical pharmacology (144, 145). Margaret Shipp and colleagues established a cohort of LBCL PDX models by implanting primary tumors underneath the renal capsule of NOD SCID *Il2rγ^null^* (NSG) mice. In total, 9 out of 28 (32%) PDX models were successfully propagated and considered a stable model. IHC characterization of these PDX models showed consistent immunophenotyping with the diagnosed LBCLs, indicating retained morphological features of the primary tumors. Furthermore, through RNA-seq analyses, the PDX models were correctly classified as ABC- (6/9 66%) or GCB-DLBCL (2/9 22%) based on their transcriptional signatures. Further genetic characterization of these PDX models revealed frequently mutated genes and chromosomal rearrangements commonly found in primary DLBCL. Lastly, the authors could show a differential sensitivity of spleen tyrosine kinase (SYK) inhibition in ABC-DLBCL PDX models, compared to GCB-DLBCL PDX models (144).

The lab of Michael Wang at MD Anderson also established a cohort of lymphoma PDX models comprised of DLBCL, MCL, MZL, BL, and FL (145). Similar to the previously mentioned PDX cohort, these PDX models also showed similar immunophenotypes and genetic profiles compared to the primary tumors. Following the characterization of the PDX models, an *in vitro* drug screen was performed with compounds that were used to treat the primary tumors in the patients. The PDX models exhibited similar response or resistance to the respective compounds as shown in patients, showcasing a robust pharmacology platform that can be enabled for personalized medicine. Moreover, the group developed primary and acquired ibrutinib-resistant PDX models to potentially reveal resistance mechanisms to BTK inhibitors. Through reverse phase protein array (RPPA) assays, they found an upregulation of PI3K pathway members, as well as BCL-2 family members following ibrutinib treatment. Follow-up combination efficacy studies in the ibrutinib-resistant PDX models with ibrutinib and idelalisib, a PI3Kδ inhibitor, ultimately overcame the ibrutinib resistance and led to significant tumor growth inhibition (145).

The two aforementioned groups faithfully established PDX platforms that can be utilized for pre-clinical pharmacology and drug discovery efforts. One possible innovation of these platforms could be to establish PDX models via intravenous transplantation of the primary tumor cells instead of subcutaneous or other orthotopic methods. This could potentially represent the disease biology in a more clinically relevant way as the tumor cells may home to organs that are commonly invaded by lymphoma cells i.e. spleen and lymph nodes. Another innovative idea might be to generate a cohort of PDX models that represent the newly classified molecular subtypes of DLBCL to further develop the idea of a genetically guided personalized medicine approach of treatment (146).

## Concluding remarks and perspectives

*In vivo* experimentation always has to be well-justified against the three Rs (reduce, refine, replace) of animal research. The passing of the FDA Modernization Act 2.0 law in December 2022 further underscores this. However, none of the currently available alternative *in vitro* model systems is capable of fully recapitulating the complex B cell activation process with the germinal center at its core, a structure central to DLBCL lymphomagenesis, nor do they faithfully model the complex lymphoma microenvironment. Therefore to date, mouse models remain a cornerstone for disease modelling and pre-clinical evaluation of potential drugs.

GEMMs, as a result of their immunocompetency and autochthonous lymphoma manifestation, represent the model system that most closely recapitulates the genetic and immunological complexity of human lymphoma. The presence of a complex tumor microenvironment, including immune cells, allows the investigation of compounds that modulate or might be influenced by the TME, including immune therapies (68, 126). However, tumors in GEMMs usually manifest with high variance after months of latency and their diagnosis ideally involves imaging methods (53, 68, 126, 147). This makes treatment experiments in GEMMs laborious and expensive. CDX/PDX models, isogenic transplantation systems of murine lymphoma cell lines or lymphoma organoids might provide more cost- and time-effective platforms for larger experimental setups with multiple treatment cohorts. Further, transplantable lymphoma cell lines obtained from GEMMs could be engineered to perform functional CRISPR screening *in vivo*, which would provide a platform to screen and test multiple therapeutic vulnerabilities in an unbiased manner.

The genesis of precision genome editing using the CRISPR/Cas9 system has enabled fast-track creation of ever-more sophisticated animal models of human lymphomas, and greatly expanded the toolkit of available preclinical models (34, 64, 148). While the generation of novel alleles has become much more efficient with these technological developments, the *ex vivo* editing of hematopoietic stem cells followed by transplantation into irradiated recipients provides a shortcut method to investigate the role of a gene-of-interest in lymphoma (104, 109, 149). By using *Cre*-dependent overexpression vectors or gRNA vectors in conjunction with a *Cre*-dependent Cas9 allele, the introduced modifications could manifest B cell-specifically.

Altogether, the choice of the experimental platform strongly depends on the research question at hand. For economic an animal welfare reasons, *in vitro* systems should be the first choice whenever appropriate. However, the complex interaction of healthy and malignant B cells with their environment requires the use of *in vivo* models in many situations. While these interactions are recapitulated by different transplantation-based systems to some extent (150, 151), autochthonous tumors developing in GEMMs most faithfully replicate human disease in terms of lymphomagenesis and tumor microenvironment and therefore will remain indispensable tools in lymphoma research in the foreseeable future.

## Author contributions

AT: Writing – original draft, Writing – review & editing. AA: Writing – original draft, Writing – review & editing. SH: Writing – original draft, Writing – review & editing. MJ: Writing – original draft, Writing – review & editing. BvT: Writing – review & editing. JH: Writing – review & editing. RF: Writing – review & editing. RJ: Writing – review & editing. SK: Writing – original draft, Writing – review & editing. HCR: Writing – original draft, Writing – review & editing. GK: Writing – original draft, Writing – review & editing.
